# Gadolinium enhancement in cervical dorsal roots in a patient with acute autonomic and sensory neuropathy: a case report

**DOI:** 10.1186/s12883-023-03186-7

**Published:** 2023-04-04

**Authors:** Du Hwan Kim, Jang Hyuk Cho, Mathieu Boudier-Revéret, Min Cheol Chang

**Affiliations:** 1 Department of Physical Medicine and Rehabilitation, Chung-Ang University, Seoul, Republic of Korea; 2 Department of Rehabilitation Medicine, Keimyung University Dongsan Hospital, Keimyung University School of Medicine, Daegu, Republic of Korea; 3 Department of Physical Medicine and Rehabilitation, University of Montreal Health Center, Montreal, Canada; 4 Department of Physical Medicine and Rehabilitation, Yeungnam University, Daegu, 42415 Republic of Korea; 5 Department of Physical Medicine and Rehabilitation, College of Medicine, Yeungnam University, 317-1 Daemyungdong, Namku, Daegu, 705-717 Republic of Korea

**Keywords:** Acute autonomic and sensory neuropathy, Magnetic resonance imaging, Diagnosis

## Abstract

**Background:**

We report an enhancement of the dorsal roots on gadolinium-enhanced cervical magnetic resonance imaging (MRI) in a patient with acute autonomic and sensory neuropathy (AASN).

**Case presentation:**

A 38-year-old woman visited our university hospital for dizziness and fainting while rising from sitting or lying down and a tingling sensation in the whole body, including her limbs, torso, and abdomen, which was sustained for 15 days. The patient had hyperalgesia in nearly her entire body and slight motor weakness in her bilateral upper and lower limbs. Autonomic dysfunction was confirmed using autonomic testing. Furthermore, the nerve conduction study showed an absence of sensory nerve action potentials in all evaluated peripheral nerves. Cervical MRI was performed 18 days after dysautonomia onset. In the axial T1-gadolinum-enhanced MRIs, enhancement in cervical ventral and dorsal nerve roots and the posterior column of the spinal cord were observed, and the axial T2-weighted MRI showed high signal intensity in the posterior column of the cervical spinal cord. Considering the clinical, electrophysiological and imaging findings, the patient was diagnosed with AASN. A total dose of 90 g (2 g/kg) of intravenous immunoglobulin was administered over 5 days. At the follow-up at 4 years after AASN symptom onset, the hyperalgesia and orthostatic hypotension symptoms improved. However, her systolic blood pressure intermittently decreased to < 80 mmHg.

**Conclusion:**

Gadolinium-enhanced MRI may facilitate the accurate and prompt diagnosis of AASN.

## Background

Acute autonomic and sensory neuropathy (AASN) is a rare disorder characterized by impaired autonomic and sensory functions, without motor impairment, that reaches its peak severity within a short period of time [1]. Although the relationship between AASN and Guillain-Barre syndrome has not yet been clarified, the progression of AASN symptoms is similar to that of Guillain-Barre syndrome [2].

AASN causes various degrees of autonomic and sensory function impairments. In some patients with AASN, mild or slight motor weakness can be combined with autonomic and sensory dysfunction [3]. In addition, there are no definite diagnostic criteria to confirm AASN. Therefore, in clinical practice, it is often ambiguous whether a diagnosis of AASN can be made, and it can be missed by clinicians.

Previous autopsy studies of patients with AASN have revealed that AASN abnormalities localize to the dorsal root ganglia, with secondary degeneration in the posterior column of the spinal cord [4, 5]. Some studies have reported a high signal intensity in the posterior column of the spinal cord on T2-weighted images [2, 6]. However, imaging abnormalities in areas other than the spinal cord have not yet been reported. Notably, the knowledge of interpreting imaging findings in patients with AASN patients could help clinicians diagnose AASN early and accurately.

In this study, we report a patient with AASN who showed enhancement of the dorsal roots on gadolinium-enhanced magnetic resonance imaging (MRI).

## Case presentation

A 38-year-old woman with no previous medical or psychological illness visited our university hospital due to dizziness and a tingling sensation in her limbs, torso, and abdomen. Approximately 18 days before her visit to our hospital, a flu-like illness developed, and she was administered oseltamivir 75 mg twice daily for 3 days. Three days after the onset of the flu-like illness, dizziness and fainting when rising from sitting or lying down suddenly occurred. Every time she stood up, these symptoms recurred and made it almost impossible for her to perform her daily life activities. In addition to dizziness and fainting, she experienced diarrhea for 3–4 days and a tingling sensation in both the hands and feet. The tingling sensation gradually spread proximally to the proximal limbs, torso, and abdomen. She also experienced mild weakness in her upper and lower limbs and reported incoordination in her bilateral upper limbs. No respiratory difficulties or voiding-related symptoms were observed.

Sensory examination revealed hyperalgesia upon light touch and pinprick in nearly her entire body including her limbs, torso, and abdomen. She had decreased sensation of vibration on both upper and lower extremities and could not discriminate between various toe positions. In addition, the motor power was determined to be 4 out of 5 on the Medical Research Council scale for muscle strength in her bilateral upper and lower limbs. The deep tendon reflexes, including biceps, triceps, knee, and ankle jerks were decreased bilaterally. She was alert. Her cerebellar signs, including the finger-to-nose test, heel-to-shin test, and Romberg test, could not be evaluated due to general weakness, dizziness, and sensory incoordination.

In autonomic testing, her blood pressure in the supine position was 132/84 mg, and her heart rate was 94 bpm. During passive upright tilt, her blood pressure fell to 91/51 mg, her heart rate increased to 107 bpm after 1 min, and she felt dizziness and a fainting sensation that was consistent with neurogenic orthostatic hypotension. In the Valsalva maneuver, no lateral phase II, overshooting phase IV, nor increased pressure recovery time (10 s) were observed, which indicated blunted baroreflex-mediated sympathetic activation. Her heart rate variation with deep breathing was reduced (expiratory inspiratory ratio, 1.06; normal range:1.3–1.7), which indicates impaired cardiovagal function.

In the nerve conduction studies performed 20 days after AASN symptom onset (Table 1), sensory nerve action potentials were absent in the bilateral median, ulnar, superficial peroneal, and sural nerves. In contrast, the motor responses on the bilateral median, ulnar, peroneal, and tibial nerves were normal.

**Table 1:** Summary of nerve conduction study

Nerve	Stimulation site	Recording site	Latency (ms)		Amplitude (μV or mV)		Conduction velocity (m/s)	
			Lt.	Rt.	Lt.	Rt.	Lt.	Rt.
Sensory nerve conduction studies								
Median	Wrist	2nd finger	NR	NR	NR	NR	NR	NR
Ulnar	Wrist	5th finger	NR	NR	NR	NR	NR	NR
Superficial peroneal	Above ankle	foot dorsum	NR	NR	NR	NR	NR	NR
Sural	Calf	ankle	NR	NR	NR	NR	NR	NR
Motor nerve conduction studies								
Median	Wrist/Elbow	APB	2.92/6.56	3.28/7.24	9.8/9.4	7.7/7.4	54.9	51.8
Ulnar	Wrist/Elbow	ADQ	2.34/5.68	2.24/5.78	8.7/7.7	7.5/6.5	57.0	56.5
Peroneal	Ankle/Knee	EDB	3.54/9.38	3.65/9.38	7.3/7.1	5.9/5.5	50.6	48.9
Tibial	Ankle/Knee	AH	3.91/12.14	3.91/12.60	14.4/12.3	10.0/8.3	43.7	42.5

*Lt.* Left, *Rt.* Right, *NR* No response, *APB* Abductor pollicis brevis, *ADQ* Abductor digiti quinti, *EDB* Extensor digitorum brevis, *AH* Abductor hallucis

The cerebrospinal fluid study at 18 days after AASN symptom onset showed an increased protein level (196.9 mg/dL) and a normal white blood cell count (1/high power filed), blood cell count (0/high power filed), and glucose level (57 mg/dL). Blood tests were negative for immunoglobulin G and immunoglobulin M of anti-GM1, anti-GD1b, and anti-GQ1b antibodies. Extensive laboratory tests to differentiate the causes of small fiber neuropathy, such as Sjögren disease, human immunodeficiency virus infection, syphilis, and pyridoxine intoxication, were negative. Chest and abdomen computed tomography for malignancy were unremarkable. Electrocardiography and anteroposterior chest radiography did not reveal any abnormalities.

**Fig. 1:**
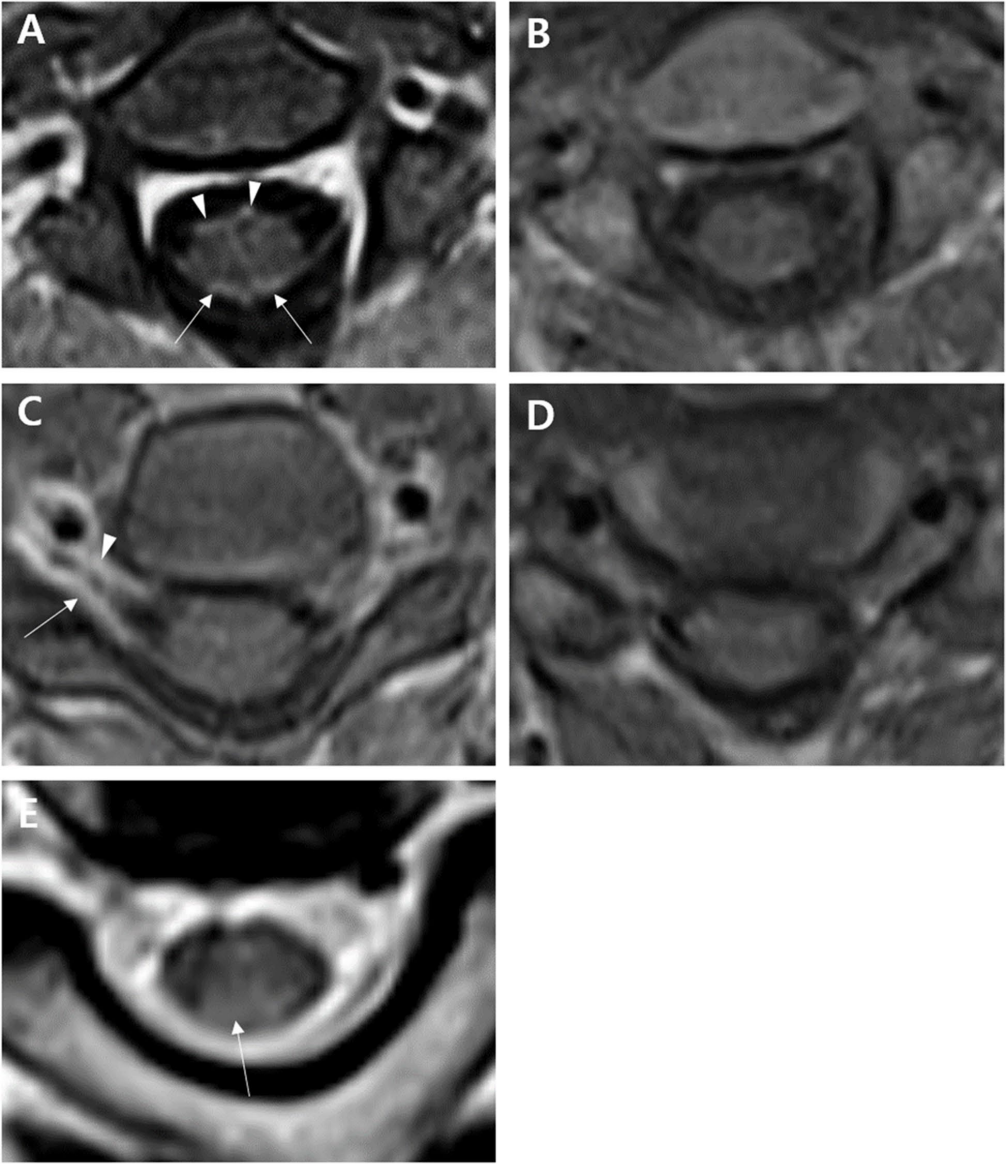
Cervical magnetic resonance imaging (MRI) taken at 18 days after the onset of acute autonomic and sensory neuropathy symptoms. Axial T1-gadolinum-enhanced MRI revealed enhancement (arrows) in cervical ventral (arrowhead) and dorsal (arrow) nerve roots at the level of C3 vertebral body (**A**) compared to non-enhanced image (**B**). At the level of C5-6 vertebra, contrast enhanced MRI also showed focal enhancement of right sixth cervical ventral (arrowhead) and dorsal nerve roots (arrow) (**C** and **D**). The T2-weighted MRI showed high signal intensity (arrow) in the posterior column of the cervical spinal cord at the C2-3 vertebral body level (**E**)

Cervical MRI was performed 18 days after AASN symptom onset. In the axial T1 enhanced MRI, enhancement of the cervical dorsal nerve roots and posterior column of the spinal cord as well as cervical ventral nerve roots was observed (Fig. 1A-D). The axial T2-weighted MRI showed a high signal intensity in the posterior column of the cervical spinal cord (Fig. 1E).

Based on the patient’s clinical presentations and the results of the physical examination, laboratory tests, and MRI, she was diagnosed with AASN.

A total dose of 90 g (2 g/kg) of intravenous immunoglobulin (IVIg) was administered over 5 days. Midodrine, fludrocortisone, and droxidopa were initiated and titrated to manage the orthostatic hypotension. Despite IVIg, symptomatic medicines, and behavior modification (e.g., slow sit-up and stand-up from lying down and sitting position) to manage orthostatic hypotension, her recurrent syncope persisted for up to 1 year after onset. In the sensory nerve conduction test performed 1.5 year after AASN symptom onset, the amplitude of the sensory nerve action potential of the bilateral median, ulnar, and sural nerves improved to approximately 5–10 μV. At a follow-up that was done 4 years after AASN symptom onset, the hyperalgesia and orthostatic hypotension had significantly reduced. However, when the patient went from a supine to standing position there was a definite drop in systolic blood pressure by about 30 mmHg with intermittent systolic blood pressure less than 80 mmHg, despite the use of midodrine (10 mg/day) and droxidopa (100 mg/day). The study was approved by the local institutional review board of Yeungnam University hospital. Written informed consent was obtained from the patient for publication of this case report and accompanying images.

## Discussion and conclusion

In this study, we describe the case of a patient with AASN. Our patient had hyperalgesia in nearly her entire body, and lesions in the cervical dorsal roots were observed on gadolinium-enhanced MRI.

Gadolinium-enhanced MRI can show inflammation in neural structures [7]. Notably, nerve enhancement on gadolinium-enhanced MRI indicates that gadolinium accumulates around the nerve tissue. Inflammation mediated by proinflammatory cytokines causes the breakdown of the blood-nerve barrier and increases vascular permeability [7]. Gadolinium exits blood vessels and is deposited around the inflammatory nerve tissue, which results in enhancement of the neural structure in contrast-enhanced MRI [7]. Previous literature reported that most patients with AASN complained of acute loss of sensory and autonomic functions after viral or bacterial infection [7]. This clinical manifestation suggests that the pathophysiology of AASN might be the same as in that of Guillain-Barré syndrome (GBS). In GBS, post-infectious immune-mediated mechanisms cause the disruption of the blood-nerve barrier leading to demyelination or axonal loss [1, 8]. Our MRI with contrast findings suggest that inflammation in the dorsal roots, together with the posterior column of the spinal cord and cervical dorsal root ganglia, is one of the causes of sensory disturbance in patients with AASN.

Even if the peripheral sensory nerve is damaged, it takes a few weeks for abnormal findings to appear in a nerve conduction study [9]. MRI with contrast is useful for the immediate detection of acute inflammatory lesions. Therefore, especially in the early stages of AASN, MRI with contrast facilitates the diagnosis of AASN. It is difficult for physicians to differentiate AASN from other mimicking disorders, such as sensory GBS, acute sensory ataxic neuropathy with antiganglioside antibodies, Sjögren disease or paraneoplastic neuronopathy because there are no specific biomarkers for AASN [1, 8]. We considered sensory GBS or acute sensory ataxic neuropathy with antiganglioside antibodies but disregarded them because of the rarity of severe autonomic dysfunction in these diseases. The imaging evidence supports the diagnosis of AASN. Hyperintensities of posterior columns on T2 weighted MRI have been reported as one of the characteristic imaging findings. This finding might be related to the postsynaptic antegrade degeneration of sensory nerves. In our patient, there was enhancement of the ventral roots even though no motor weakness was reported. In typical GBS, only ventral root enhancement is commonly involved rather than both ventral and dorsal root enhancement [10]. In this case, enhancement of the ventral roots may reflect sympathetic dysfunction rather than motor weakness because the ventral root contains the autonomic efferents.

In conclusion, enhanced MRI facilitates the accurate and prompt diagnosis of AASN. To confirm our enhanced MRI findings, further studies involving a larger number of patients with AASN should be conducted.

## Data Availability

The datasets used during the current study are available from the corresponding author on reasonable request.

## Abbreviations

AASN: Acute autonomic and sensory neuropathy
MRI: Magnetic resonance imaging
IVIg: Intravenous immunoglobulin
